# Single-Dose Vaccination of Recombinant Chimeric Newcastle Disease Virus (NDV) LaSota Vaccine Strain Expressing Infectious Bursal Disease Virus (IBDV) VP2 Gene Provides Full Protection against Genotype VII NDV and IBDV Challenge

**DOI:** 10.3390/vaccines9121483

**Published:** 2021-12-15

**Authors:** Qilong Qiao, Mingzhen Song, Congcong Song, Yihang Zhang, Xiangdong Wang, Qing Huang, Baiyu Wang, Panpan Yang, Shiyi Zhao, Yongtao Li, Zeng Wang, Jun Zhao

**Affiliations:** College of Veterinary Medicine, Henan Agricultural University, Zhengzhou 450046, China; QilongQiao@163.com (Q.Q.); mingzhenSong123@163.com (M.S.); songcongcong1998@163.com (C.S.); yihangzhang0112@163.com (Y.Z.); WXD21203133@163.com (X.W.); qinghuang202111@163.com (Q.H.); sherrywang828@hotmail.com (B.W.); Yangpanpan617@163.com (P.Y.); zsyi1998@163.com (S.Z.); yongtaole@126.com (Y.L.); wzzxz20140105@126.com (Z.W.)

**Keywords:** genotype VII Newcastle disease virus, infectious bursal disease virus, VP2 gene, chimeric LaSota vaccine strain

## Abstract

Newcastle disease virus (NDV) and infectious bursal disease virus (IBDV) are the two most important and widespread viruses causing huge economic losses in the global poultry industry. Outbreaks of genotype VII NDV and IBDV variants in vaccinated poultry flocks call for genetically matched vaccines. In the present study, a genetic matched chimeric NDV LaSota vaccine strain expressing VP2 gene of IBDV variant, rLaS-VIIF/HN-VP2 was generated for the first time, in which both the F and HN genes of LaSota were replaced with those of the genotype VII NDV strain FJSW. The cleavage site of the FJSW strain F protein in the rLaS-VIIF/HN-VP2 genome was mutated to the avirulent motif found in LaSota. Expression of IBDV VP2 protein was confirmed by western blot. The rLaS-VIIF/HN-VP2 maintained the efficient replication ability in embryonated eggs, low pathogenicity and genetic stability comparable to that of parental LaSota virus. One dose oculonasal vaccination of one-week-old SPF chickens with rLaS-VIIF/HN-VP2 induced full protection against genotype VII NDV and IBDV lethal challenge. These results indicate that the rLaS-VIIF/HN-VP2 is a promising bivalent vaccine to prevent infections of IBDV and genotype VII NDV.

## 1. Introduction

Newcastle disease virus (NDV), a member of the genus *Orthoavulavirus* of subfamily *Avulavirinae*, family *Paramyxoviridae*, is the causative agent of Newcastle disease which is a highly contagious and devastating avian disease causing severe economic losses in the global poultry industry [1]. Although all NDV strains belong to a single serotype, there is antigenic and genetic diversity among NDV isolates [2]. NDV strains are generally classified into velogenic, mesogenic, and lentogenic pathotypes based on conventional in vivo pathogenicity indices such as the mean death time (MDT) performed in 9- to 10-day-old chicken embryos and the intracerebral pathogenicity index (ICPI) in one-day-old chicks [3,4]. Classification based on the sequence and phylogenetic analysis of the F gene further divides NDV strains into class I, with only one genotype, and class II, having up to 18 genotypes [5]. Most of the popular commercially available ND vaccines are of genotypes I and II whilst virulent NDVs are grouped in genotypes III to XVIII [6]. Since the 1990s, ND caused by genotype VII NDV strains were frequently reported in China and other regions, and molecular epidemiology data have indicated that genotype VII NDV is the predominant virus strain currently circulating in Asia [7,8,9,10]. Genetic distance analysis based on the full-length F and HN genes showed that genotype VII NDV isolates had high genetic variations with genotype II LaSota vaccine [11]. Accumulating data indicated that genotype-matched vaccines provide better protection against genotypes VII NDV infection than the LaSota vaccine [11,12]. Currently, most of the reported genotype VII-matched ND vaccine candidates were generated by modifying the sequence encoding the fusion protein (F) cleavage site of genotype VII NDV from virulent polybasic (RRQKRF) to avirulent monobasic (GRQGRL) motif using reverse genetics technique [13,14]. Although the genotype matched ND attenuated live vaccines are superior than traditional live vaccines, there is a concern that the mutated F protein cleavage site might revert to wild type through homologous recombination with wild type virus when used in the field. An alternative strategy for producing genotype matched vaccines is to replace the F and HN genes of a current commercial vaccine strain, such as LaSota [15,16,17]. 

Infectious bursal disease (IBD) is a highly contagious, immunosuppressive disease of young chickens caused by infectious bursal disease virus (IBDV) which is a pathogen of major economic importance in the poultry industry worldwide [18]. IBDV infection results in the destruction of B cells in the bursal of Fabricius, and consequently immunosuppression, which leads to vaccination failures and susceptibility to other infections and diseases [19]. IBDV belongs to the genus *Avibirnavirus* in the family *Birnaviridae*. The genome of the IBDV is bi-segmented and divided into segments A and B. The segment A encodes four proteins (VP2, VP3, VP4 and VP5). Among them, the VP2 is the major structural protein of IBDV and host-protective immunogen [2]. The VP2 protein contains major epitopes responsible for eliciting neutralizing antibodies. Protection against IBDV has been induced by recombinant VP2 protein expressed in different systems [20]. Currently, inactivated whole IBDV vaccines, VP2-based subunit vaccines, and live attenuated vaccines were mainly used for the prevention and control of IBD. In recent years, atypical IBD caused by novel IBDV variants with severe immunosuppression has brought new threats to the poultry industry and has caused considerable economic losses [21,22,23,24,25]. Novel variants of IBDV resulted in severe atrophy of the bursa of Fabricius in chickens immunized with vaccines against very virulent IBDV (vvIBDV) and the antigenic mismatch between novel variant IBDV and vvIBDV has been confirmed [24]. The typical amino acid residues of the variant IBDVs 222T, 249K, 286I, and 318D in the hypervariable region (HVR) of VP2 were observed in a representative Chinese IBDV variant SHG19. Interestingly, one IBDV isolate TL in our lab also has the characteristic aa residues 249K and 286I, and can cause severe atrophy of the bursa of Fabricius and typical IBD. However, the aa identity of VP2 between SHG19 and TL strain was only 93.9%. A recent study has demonstrated that a novel IBDV variant suppresses the ND vaccination in broiler and layer chickens [26]. Therefore, it is urgent to develop new vaccines against genotype VII NDV and variant IBDV.

Advances in NDV molecular biology and reverse genetics have allowed us to engineer genotype-matched NDV vaccines. Different pathotypes of NDV strains have been developed as vectors to express IBDV VP2 and other immunogens [27,28,29,30,31,32,33,34]. A recombinant NDV vaccine strain F expressing the VP2 gene of vvIBDV, rNDV F/VP2 induced both humoral and cell mediated immunity, and was able to confer complete protection against vvIBDV challenge and 80% protection against virulent NDV challenge [27]. After in-ovo vaccination, a chimeric NDV LaSota virus with the L gene of Clone-30 expressing the VP2 protein of vvIBDV, rLaC30L-VP2 provided 100% protection against a lethal NDV challenge and resistance against vvIBDV challenge in a significant amount of animals [28]. A recombinant LaSota expressing soluble form of the VP2 protein of a variant IBDV strain GLS-5 provided 90% protection against virulent NDV strain Texas GB and virulent IBDV GLS-5 strain [29]. In the above-mentioned studies, the F and HN antigens are natively presented on the administered virions, while the VP2 protein was not incorporated into the virions. Considering the safety and efficacy of the current genotype VII NDV-matched vaccine candidates, in this study, a genotype-matched NDV-vectored bivalent vaccine candidate against infection of virulent IBDV variant and genotype VII NDV was developed for the first time. The vaccine candidate was constructed by using chimeric LaSota as a vector in which both the F and HN genes of LaSota were replaced with those of the circulating and highly virulent Chinese genotype VII NDV strain. The VP2 gene inserted in the vector was obtained from the novel variant IBDV. The efficiency of the rLaS-VIIF/HN-VP2 was assessed by oculonasal vaccination of one-week-old SPF chickens.

## 2. Materials and Methods

### 2.1. Cells, Viruses, and Ethics Statement

BHK-21 (Baby hamster kidney) cells (ATCC, Manassas, VA, USA) were cultured in Dulbecco’s modified Eagle medium (DMEM) (Cytiva, Logan, UT, USA) supplemented with 10% fetal bovine serum (FBS) (Gibco, Waltham, MA, USA) and maintained at 37 °C with 5% CO_2_. Recombinant vaccinia virus vTF7-3 expressing T7 RNA polymerase was grown in BHK-21 cells. A genotype VII NDV velogenic strain FJSW (MDT < 60 h; ICPI > 1.50) isolated from broiler chickens in Fujian province, China in 2017 was propagated in 10-days-old specific-pathogen-free (SPF) embryonated chicken eggs through allantoic inoculation. Virulent IBDV isolate TL was grown in 10-days-old specific-pathogen-free (SPF) embryonated chicken eggs inoculated through the chorioallantoic membrane (CAM) route. All of the viruses are available at Institute of Poultry Diseases, Henan Agricultural University, China. The Ethics and Animal Welfare Committee of Henan Agricultural University, China, reviewed all experiment procedures and approved this project (HENAU-SYXK-20210512-01).

### 2.2. Plasmid Construction and Recovery of Recombinant Virus

The Plasmid pNDFL carrying the full-length cDNA of the NDV vaccine strain LaSota has been described previously [35]. Plasmid pNDFL-VII-F/HN was generated by replacing the F and HN genes in pNDFL with those of the circulating and highly virulent Chinese genotype VII NDV strain FJSW. The sequence encoding the F protein cleavage site of FJSW in pNDFL-VII-F/HN was modified from virulent polybasic (RRQKRF) to avirulent monobasic (GRQGRL) by overlapping PCR. The helper plasmid pCIneo-NP-P-L expressing the NP, P, and L proteins of the LaSota strain was constructed by cloning the corresponding genes into a pCI-neo expression vector (Promega, Madison, WI, USA). The NP, P, and L proteins expressed by the helper plasmid form a ribonucleoprotein complex with the viral RNA genome to support viral genome transcription and replication [2]. The codon-optimized coding sequence of the VP2 gene based on a novel IBDV variant SHG19 (GenBank accession No. MN393076) was synthesized and cloned into pUC57 vector (GenScript, Nanjing, China) to generate plasmid pUC-VP2. To facilitate insertion of VP2 gene of a novel IBDV variant between the P and M genes in the plasmid pNDFL-VII-F/HN, the VP2 gene was amplified by PCR using forward primer 5′-GCTCTTCAAGC**GCCACC**ATGACAAACCTGCAAGATC-3′ and reverse primer 5′-TGAGGAAGAGCTTACCTCCTTATAGCCCGG-3′, each containing a SapI site (underlined) and a Kozak sequence upstream of the start codon (labeled in bold). The amplified VP2 gene was cut by SapI and cloned into the pGEM-PM shuttle vector in which an extra gene end (GE) and gene start (GS) box following with two SapI sites were inserted into the non-coding region between P and M gene of the LaSota strain. The resulting pGEM-PM-VP2 and pNDFL-VII-F/HN plasmids were digested with ApaI and PmlI, and the liberated fragments were ligated together, resulting in pNDFL-VII-F/HN-VP2.

Recombinant NDV was recovered using standard protocols as described previously [35]. Briefly, BHK-21 cells grown to 60% confluence in six-well culture plates were infected with vTF7-3 for 1 h at 37 °C. Subsequently, the cells were co-transfected with 2.0 μg of pNDFL-VII-F/HN-VP2 and pCIneo-NP-P-L using Lipofectamine^®^ 3000 (Thermo Fisher Scientific, Waltham, MA, USA). The supernatant was collected after 72 h, passed through a 0.22 μm-pore-size filter and inoculated into 10-day-old chicken embryo by allantoic cavity route to amplify the rescued virus. The allantoic fluid was harvested after 94 h. Hemagglutination assay (HA) was performed to verify the rescued rLaS-VIIF/HN-VP2. The HA assay was performed in a 96-well microtiter plate by mixing the allantoic fluids with 1% chicken red blood cells and incubating at 37 °C for 20 min. The successful rescue of the virus was indicated by the hemagglutination effects. 

### 2.3. Characterization of Recombinant Viruses

To assess the pathogenicity of the rLaS-VIIF/HN-VP2, in vivo pathogenicity indices, such as the MDT and the ICPI, were determined in 9- to 10-day-old chicken embryos and in 1-day-old chicks, respectively [36]. For NDV, the MDT value greater than 90 h and the ICPI value less than 0.7 were defined as “lentogenic” according to World Organization for Animal Health [1]. The stability of the rLaS-VIIF/HN-VP2 was determined by detecting the inserted VP2 gene in different passages of the rLaS-VIIF/HN-VP2 using RT-PCR with forward primer 5′-GGAAAATCAAGCGCCTTGCTC-3′ and reverse primer 5′-GACGATCGGAAATGCTAACAGG-3′ and sequencing the amplified products. The growth kinetics of the parent LaSota and the recombinant virus in SPF eggs, and 50% embryo infective dose (EID_50_) were measured in nine-day-old chicken embryos as described previously [37]. Expression of the VP2 protein in rLaS-VIIF/HN-VP2 was examined by Western blot using IBDV-specific monoclonal antibody (mAb) against the VP2 protein. Briefly, the allantoic fluid from rLaS-VIIF/HN-VP2-and LaSota-inoculated SPF eggs were harvested. Protein samples were resolved on 4–20% SDS-PAGE, and then blotted onto a nitrocellulose membrane. After blocking in 5% non-fat dry milk in PBS containing 0.1% Tween-20 (PBST) at 4 °C overnight, the VP2 protein was detected by 1:500 diluted anti-IBDV VP2 mAb and 1:3000 diluted goat anti-mouse IgG-HRP (Proteintech Group, Rosemont, IL, USA). After washing three times, the protein bands were visualized using ECL substrate (Thermo Fisher Scientific, Waltham, MA, USA) in the dark.

### 2.4. Immunization and Challenge Experiment

The immunogenicity and protective efficiency of rLaS-VIIF/HN-VP2 were determined in one-week-old SPF chickens. Twenty chickens were vaccinated via the oculonasal route with 100 μL (10^7^ EID_50_) of the rLaS-VIIF/HN-VP2. Twenty chickens were inoculated with the same volume of allantoic fluid from SPF eggs as a placebo control through the same route. Twenty chickens were used as uninfected controls. Four weeks post-immunization half of chickens from each immunized group were oculonasally challenged with the genotype VII NDV velogenic strain FJSW (1 × 10^5^ 50% embryo lethal doses [ELD_50_] per chicken) and 1 × 10^4^ ELD_50_/chicken of IBDV TL strain, respectively. Chickens were observed daily for seven days after challenging. 

Serum samples were collected from chickens before immunization and each week post-immunization to determine the level of hemagglutination inhibition (HI) antibody titers against NDV by conventional HI assay and the antibody response against IBDV by a commercial ELISA kit.

Lung, trachea, spleen, proventriculus, duodenum, cecal tonsil, and bursa of Fabricius tissue samples of chickens in each group were collected from dead chickens during the experiment or euthanized chickens at the end of experiment. Bursa samples from all chickens challenged with IBDV were weighed for comparison of bursa-to-body weight ratio (BBWR). The BBWR = bursal weight/body weight × 1000. The gross lesions of organs were evaluated. Each organ was cut into two parts (one part for microscopic lesion analysis, another part for viral load measurement by a SYBR Green I real-time PCR (RT-qPCR)).

Oropharyngeal and cloacal swabs collected daily from chickens challenged with NDV and cloacal swabs from chickens challenged with IBDV were used to evaluate NDV and IBDV sheddings by RT-PCR, respectively. Primers NDV-F: 5′-GGAAGATCAAACGCCTTGC-3 and NDV-R: 5′-GACAATCGGGAATGCTAACAGG-3′ were used for detecting the shedding of for genotype VII NDV. Templates of the challenge genotype VII NDV will produce a 325 bp fragment and the rLaS-VIIF/HN-VP2 will produce a 1681bp fragment, respectively. The viral shedding of IBDV was detected by RT-PCR using primers VP1-F 5′-AGTCCACAGGCGCGAAGCA-3′ and VP1-R 5′-GATGGAGCTGACCATATGTT-3′. The template of the challenge IBDV will produce a 1000 bp fragment.

### 2.5. Hemagglutination Inhibition Assay (HI)

The immune response against NDV induced by the live rLaS-VIIF/HN-VP2 vaccine was evaluated by a HI [38]. The HI was performed against four hemagglutination units of the genotype VII NDV isolate FJSW. 

### 2.6. ELISA for Detecting Antibody against IBDV

The antibody response against IBDV generated by the live rLaS-VIIF/HN-VP2 was evaluated by using the ProFLOK IBD PLUS Ab (Zoetis, Synbiotics, Parsippany-Troy Hills, NJ, USA) ELISA kit according the manufacturer’s instructions. ProFLOK™ IBD PLUS Ab is a rapid screening ELISA for the detection of pre- and post-vaccination IBD antibodies in chickens. The kit contains an IBDV antigen produced in vivo and extracted from infected bursae of Fabricius including a purification step. Briefly, 1:100 diluted sera were transferred into the wells of IBDV viral particle and VP2 protein coated microplate in duplicates and incubated for 30 min at room temperature. After three times washing with 1× wash solution, 100 μL of 1× Conjugate Solution was added to each well, following by incubation for 30 min at RT and three times washing with 1× wash solution. Subsequently, 100 μL of Substrate were added to each test well and the optical density (OD) was measured at 405 nm after adding 100 μL/well of 1× Stop Solution. A sample to positive (S/P) ratio was calculated by subtracting the average normal control OD from each sample OD and dividing the difference by the corrected positive control which is the average positive control OD subtracted by the average normal control OD. An ELISA titer for IBD was calculated the suggested equation: Log_10_Titer = (1.172 × Log_10_S/P) + 3.614. Then Titer = antilog of Log_10_Titer.

### 2.7. SYBR Green I Quantitative Real-Time Polymerase Chain Reaction (RT-qPCR)

The IBDV viral loads were determined by the SYBR Green I RT-qPCR performed with a CFX96 Real-Time System (Bio-Rad, California, US) The IBDV VP1 gene was used as an indicator for the presence of IBDV RNA. The forward and reverse primers were VP1-qF: 5′-AGTCCACAGGCGCGAAGCA-3′ and VP1-qR: 5′-CTTTGCCAGTCGACTAGG-3′. The genotype VII NDV HN gene was used as an indicator for the presence of genotype VII NDV RNA. The forward and reverse primers were HN-qF: 5′-GCAGAGACCACTCACACTCACA-3′, HN-qR: 5′-TGCAGGACTTCCGATTTTGGGTG-3′. The RT-qPCR system includes 10 μL of 2× ChamQ Universal SYBR qPCR Master Mix (Vazyme Biotech, Nanjing, China), 1 μL of each primer (10 μM) and 1 μL of the plasmid template in a total volume of 20 μL. The reaction conditions were as follow: predenaturation at 95 °C for 30 s and amplification for 40 cycles at 95 °C 10 s and 60 °C 15 s. The amplified 138-bp IBDV VP1 gene fragment and the 129-bp NDV HN gene fragment were purified and cloned into the pMD18-T vector (TaKaRa Biotech, Dalian, China) to prepare a standard recombinant plasmid, respectively. Serial dilutions of the standard plasmid from 10^9^ to 10^3^ copies/μL were used to optimize the RT-qPCR reaction and to establish the standard curve by CFX Maestro software (Bio-Rad, Hercules, CA, USA). The final concentration was calculated as copy numbers per milligram tissue samples. Results are presented at means ± standard deviation.

### 2.8. Statistical Analysis

Statistical analysis was performed using the student *t* test in the GraphPad Prism 8.3.0 software package. The normality of the data was assessed by the D’Agostino-Pearson normality test. Unpaired and parametric *t* tests were performed between the mean values of viral loads in organs of vaccinated and unvaccinated groups. Significant difference was determined as ** *p* < 0.01.

## 3. Results

### 3.1. Generation and Characterization of Chimeric LaSota Expressing IBDV VP2

**Figure 1:**
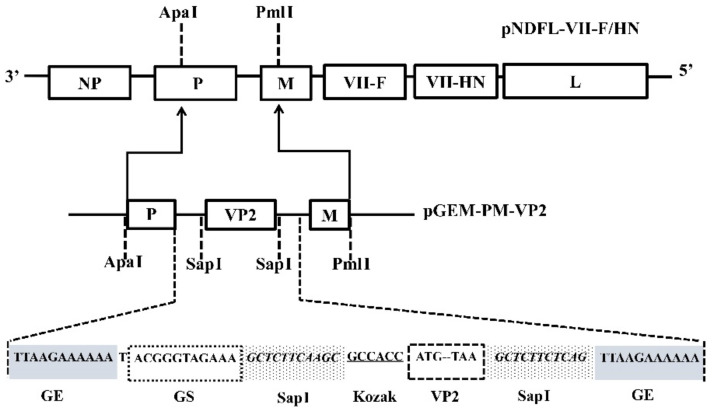
Diagram of cDNA construct for recombinant chimeric LaSota expressing IBDV VP2 gene: The VP2 gene of a novel IBDV variant SHG19 strain was inserted between P and M genes in the pGEM-PM plasmid. The generated pGEM-PM-VP2 plasmid was cut with ApaI and PmlI, and the fragment containing VP2gene was cloned into ApaI and PmlI digested pNDFL-VII-F/HN to make pNDFL-VII-7F/HN-VP2 construct.

To generate a chimeric LaSota with genotype VII NDV F and HN genes expressing IBDV VP2 gene, the VP2 gene from a novel IBDV Chinese variant strain SHG19 was inserted between the P and M genes of the full-length cDNA of chimeric LaSota to get the plasmid pNDFL-VII-F/HN-VP2 (Figure 1), in which the F and HN genes was replaced with those of the circulating and highly virulent Chinese genotype VII NDV strain FJSW. 

**Figure 2:**
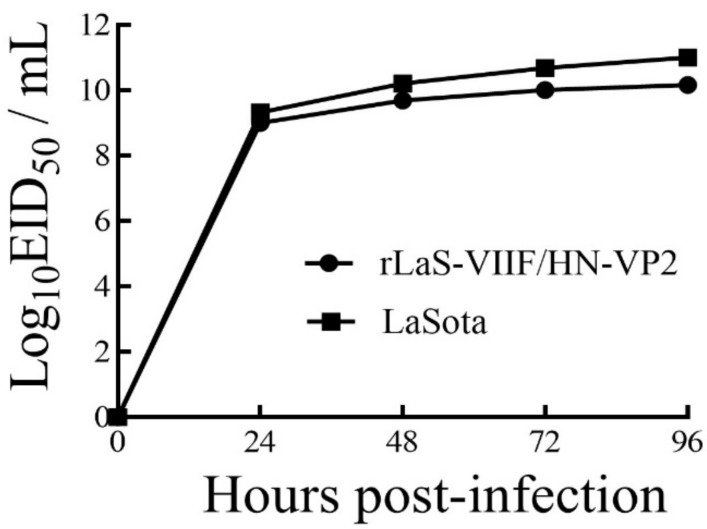
Growth kinetics of recombinant rLaS-VIIF/HN-VP2 and LaSota in SPF eggs: 10^2^ EID_50_ (50% egg infections dose, EID_50_) of NDV LaSota strain and the 25th passage rLaS-VIIF/HN-VP2 was inoculated into the allantoic cavity of 10-day-old embryonated SPF eggs. The allantoic fluid was harvested at 12, 24, 36, 48, 60, 72, and 96 h post-infection (hpi). The EID_50_ of the virus at each time point was measured.

The recombinant virus rLaS-VIIF/HN-VP2 was rescued by co-transfecting the pNDFL-VII-F/HN-VP2 with a supporting plasmid pCIneo-NP-P-L into BHK-21 cells pre-infected with vTF7-3 which expresses T7 RNA polymerase. The MDT value of The MDT and ICPI of the rLaS-VIIF/HN-VP2 was 134 h and 0, respectively. It is proved that the rLaS-VIIF/HN-VP2 maintains the low pathogenicity as LaSota vaccine strain. Moreover, a similar replication pattern in chicken embryo was observed between rLaS-VIIF/HN-VP2 and the parent LaSota strain, and the titer of the 25th passage rLaS-VIIF/HN-VP2 reached up to 10^10.16^ EID_50_/mL (Figure 2).

**Figure 3:**
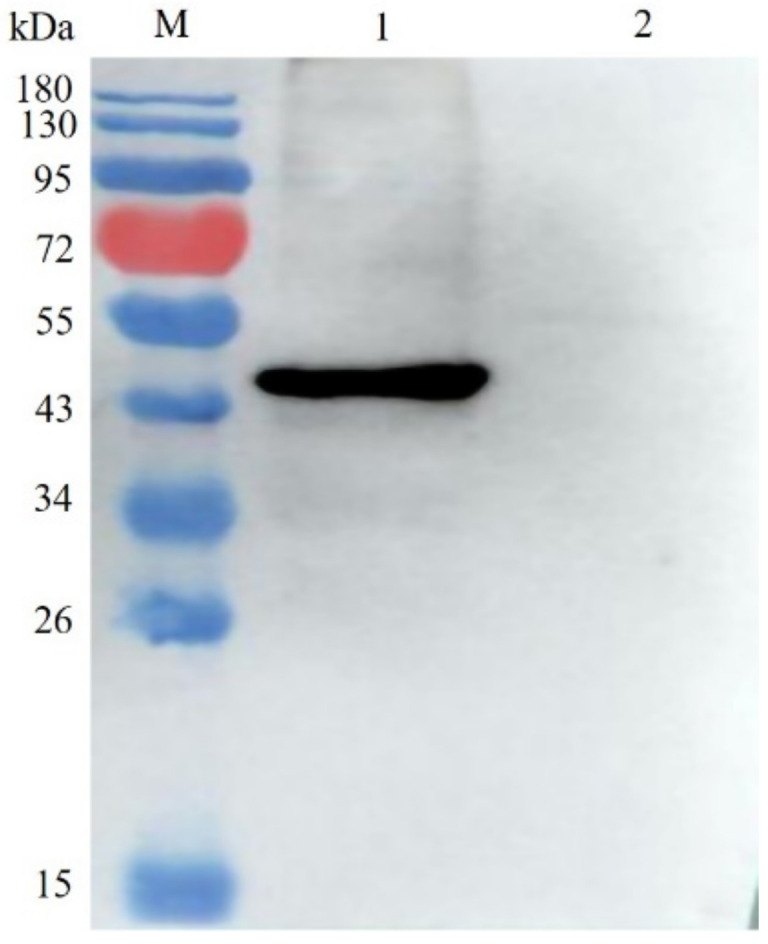
Western blotting analysis of IBDV VP2 protein expression by recombinant virus: IBDV VP2 protein expressed by the rLaS-VIIF/HN-VP2 was detected by western blot using anti-IBDV VP2 monoclonal antibody. M. Pre-stained protein molecular weight standards; Lane 1. rLaS-VIIF/HN-VP2-infected chicken embryo allantoic fluid; Lane 2. LaSota LaSota-infected chicken embryo allantoic fluid.

The expression of VP2 protein by the rLaS-VIIF/HN-VP2 was verified by Western blotting. The results in Figure 3 showed that the IBDV VP2 protein was successfully expressed with expected size (41 kDa). The results of RT-PCR and western blot demonstrated the stability of rLaS-VIIF/HN-VP2 (data not shown). 

### 3.2. Immunogenicity and Protective Efficacy of Recombinant NDV

**Figure 4:**
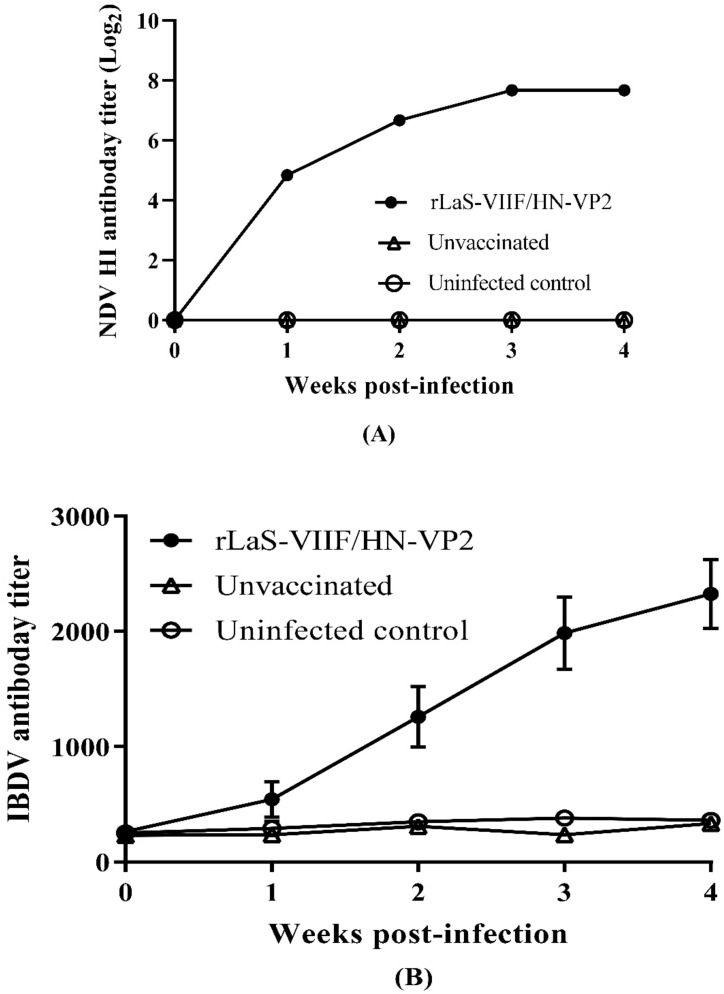
Antibody responses against genotype VII NDV and IBDV in chickens immunized oculonasally with live rLaS-VIIF/HN-VP2: (**A**) HI antibody titers against genotype VII NDV. Mean HI titer plus standard deviation of each group were show; (**B**) ELISA antibody titers against IBDV. Mean antibody titers plus standard deviation are shown.

After one dose oculonasal vaccination of chickens with live rLaS-VIIF/HN-VP2, the antibody responses against NDV and IBDV were determined by HI assay and ELISA, respectively. The results showed that one dose live rLaS-VIIF/HN-VP2 vaccination generated remarkable immune responses to both NDV and IBDV (Figure 4A,B). The peak HI antibody titer against genotype VII NDV reached 2^8^, and the peak ELISA titer against IBDV reached more than 2000 at week four post-immunization. 

**Figure 5:**
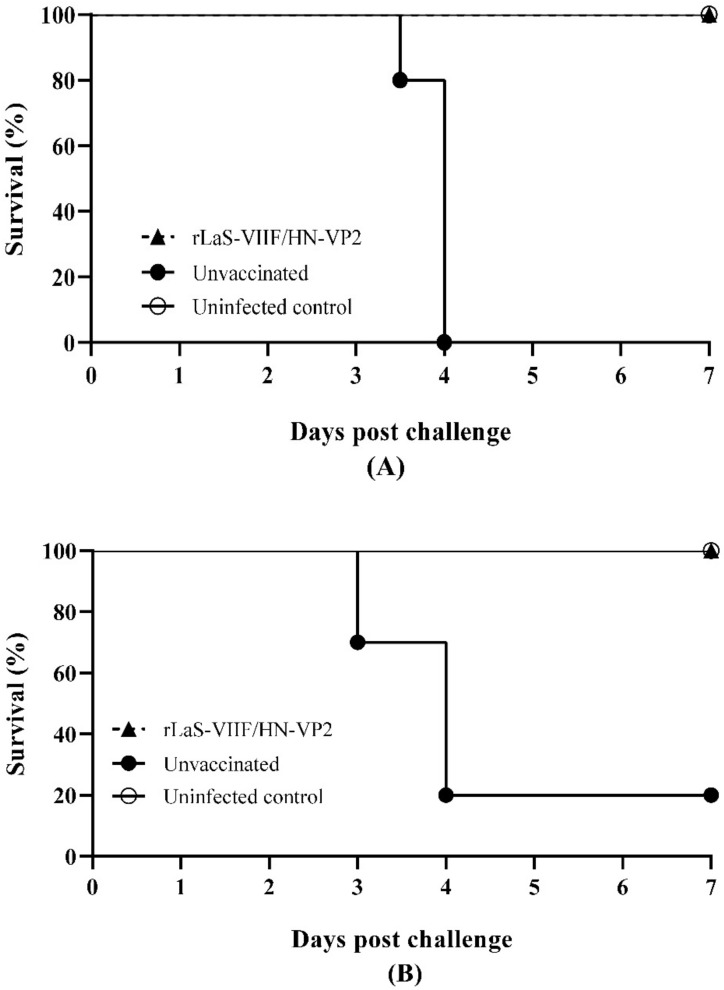
Survival rates of chickens post genotype VII velogenic NDV and variant IBDV strain challenge: Half of the chickens in rLaS-VIIF/HN-VP2 vaccinated and placebo control group were oculonasally challenged with 10^5^ ELD_50_/chicken of velogenic genotype VII NDV isolate FJSW (**A**) and 10^4^ ELD_50_/chicken of IBDV TL strain (**B**), respectively.

Four weeks post immunization, half of the chickens in live rLaS-VIIF/HN-VP2 immunized and placebo control groups were challenged oculonasally with 10^5^ ELD_50_/chicken of the genotype VII NDV velogenic strain FJSW and 10^4^ ELD_50_/chicken of IBDV TL strain, respectively. As shown in Figure 5, chickens vaccinated with live rLaS-VIIF/HN-VP2 were fully protected against a lethal dose of genotype VII NDV. However, placebo control chickens ended with 100% mortality on day 4 after the genotype VII NDV challenge (Figure 5A). Vaccination with live rLaS-VIIF/HN-VP2 vaccine provided full protection against virulent IBDV challenge. Unvaccinated chickens challenged with virulent IBDV became listless on day two post challenge and ended with 80% mortality (Figure 5B). 

### 3.3. Single-Dose Live rLaS-VIIF/HN-VP2 Vaccination Reduced Clinical Signs of Disease, Gross and Histological Lesions, Viral Shedding and Viral Loads

**Figure 6:**
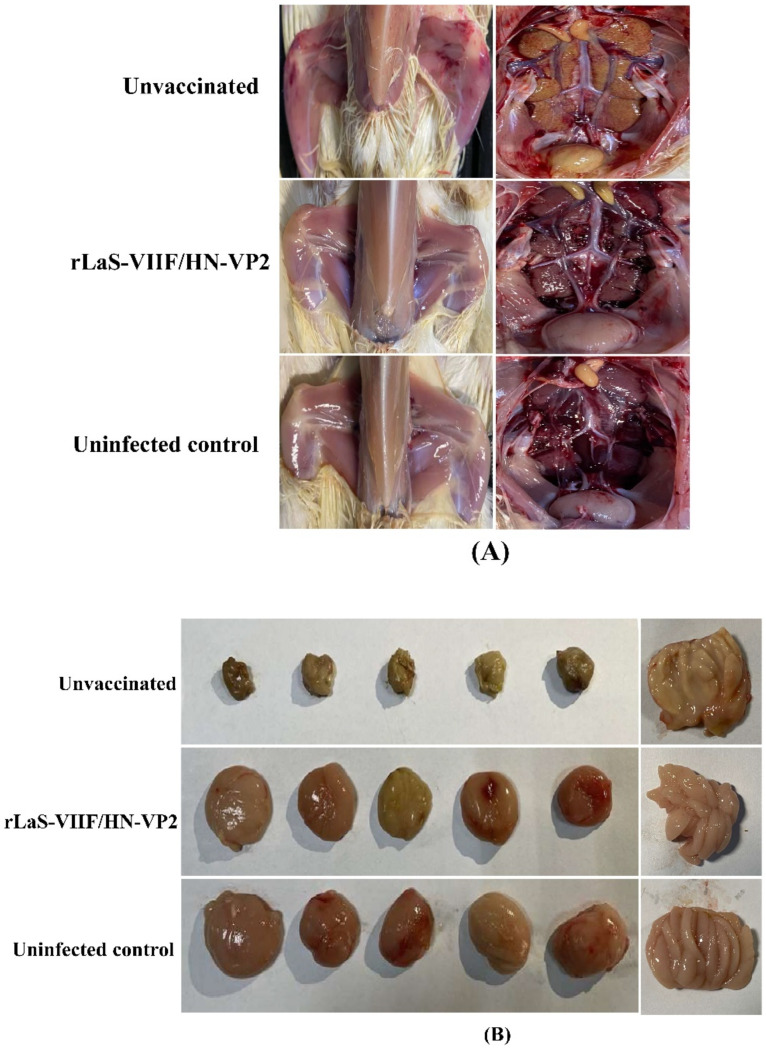
Representative postmortem examinations of chickens challenged with virulent IBDV TL strain: (**A**) Postmortem examination of unvaccinated chickens showed hemorrhages in the thigh, swelling and paleness of the kidneys, and urate deposits in the ureters. (**B**) The bursa of Fabricius of all unvaccinated chickens appeared severe atrophy and had a gelatinous yellowish transudate covering the mucosal surface. Chickens immunized with the live rLaS-VIIF/HN-VP2 did not show obvious gross lesions.

After virulent IBDV TL strain challenge, unvaccinated chickens appeared listless and discharged white feces on day two post challenge. The onset of mortality was seen on day three post challenge. Postmortem examination of unvaccinated chickens showed hemorrhages in thigh, swelling and paleness of kidneys and urate deposits in the ureters. The bursa of Fabricius of all unvaccinated chickens appeared severe atrophy and had a gelatinous yellowish transudate covering the mucosal surface (Figure 6A,B). 

**Figure 7:**
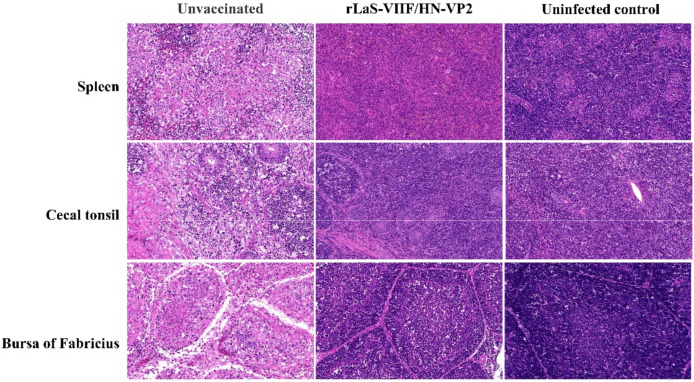
Typical microscopic lesions of lymphoid organs from chickens after virulent IBDV TL strain challenge. The microscopic lesions in unvaccinated chickens occurred primarily in the lymphoid tissues. There was severe depletion, degeneration, and necrosis of lymphocytes in the medullary area of bursal follicles. The main lesions in spleen and cecal tonsil included lymphoid necrosis in the germinal follicles and the periarteriolar lymphoid sheath. No obvious tissue damages were seen in the lymphoid organs of chickens vaccinated with the live rLaS-VIIF/HN-VP2 and uninfected controls (H&E stain, original magnification 400×).

Bursa to body weight ratio of unvaccinated chickens was significantly lower than those of rLaS-VIIF/HN-VP2 immunized and the uninfected control chickens (*p* = 0.0001). The microscopic lesions in unvaccinated chickens occurred primarily in the lymphoid tissues with the most severe lesions in the bursa of Fabricius. There was severe depletion, degeneration, and necrosis of lymphocytes in the medullary area of bursal follicles. The main lesions in spleen and cecal tonsil included lymphoid necrosis in the germinal follicles and the periarteriolar lymphoid sheath (Figure 7). The rLaS-VIIF/HN-VP2 immunized and uninfected control chickens remain healthy and did not show obvious gross and microscopic lesions.

**Figure 8:**
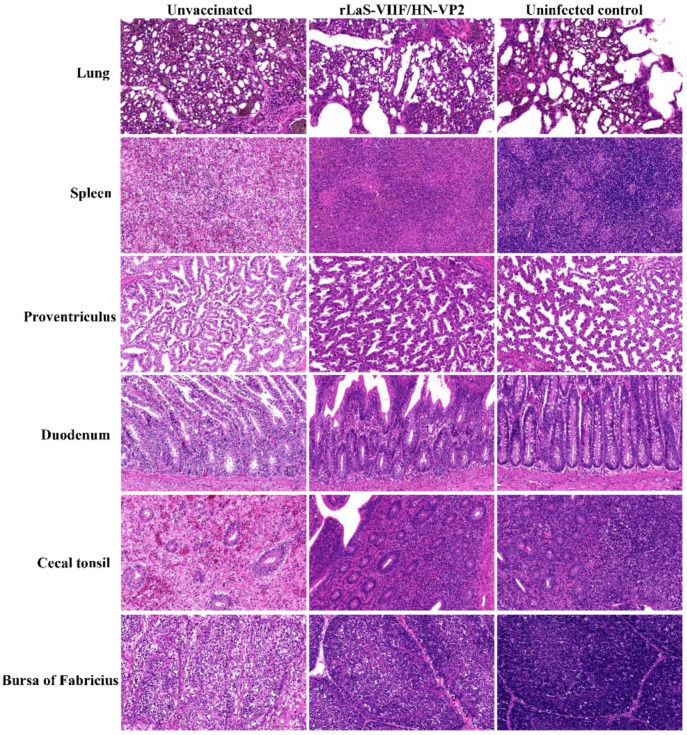
Typical microscopic lesions of in different organs from chickens after virulent genotype VII NDV FJSW challenge: The histological lesions presented by the unvaccinated chickens included interstitial chronic inflammatory infiltrate in lung; necrosis with lymphoid depletion of spleen, cecal tonsil and bursa of Fabricius; necrosis of epithelium of intestine and proventriculus. Organs of chickens vaccinated with the live rLaS-VIIF/HN-VP2 and the uninfected controls did not exhibit obvious microscopic lesions. (H&E stain, original magnification 400×).

After the virulent genotype VII NDV challenge, unvaccinated chickens became listless and depressed, gasping for air on day two post challenge. The diseased birds discharged green and watery feces. The onset of mortality was seen at 84 h post challenge. Gross lesions, including necrosis and hemorrhage of the proventriculus, cecal tonsils and enlarged, mottled spleen with pinpoint areas of necrosis were seen for dead birds in the unvaccinated group. Chickens from the liver LaS-VIIF/HN-VP2 vaccinated group and uninfected control remained clinical health and did not present obvious gross lesions. The histological lesions observed in the sick or dead chickens included interstitial chronic inflammatory infiltrate in lung; necrosis with lymphoid depletion of the spleen, cecal tonsil, and bursa of Fabricius; necrosis of epithelium of the intestine; and proventriculus. In contrast, chickens vaccinated with the live rLaS-VIIF/HN-VP2 and the uninfected controls did not exhibit obvious microscopic lesions (Figure 8).

**Figure 9:**
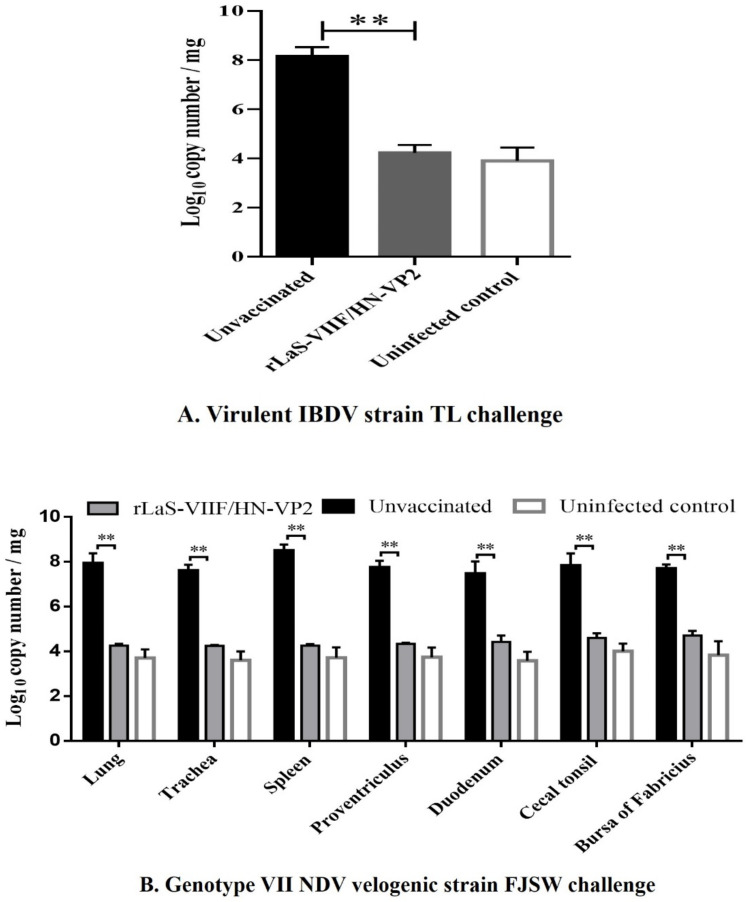
Viral loads in different tissues of chickens challenged with virulent IBDV TL strain and genotype VII NDV FJSW strain. Viral loads in the lung, trachea, spleen, duodenum, proventriculus, cecal tonsil, and bursa of Fabricius tissue samples were quantitated by a SYBR Green I quantitative real-time PCR. (**A**) IBDV viral loads in bursa of Fabricius tissues were quantitated by using IBDV VP1 as an indicator for the presence of viral RNA. (**B**) Genotype VII NDV viral loads in different tissues were determined using genotype VII NDV HN gene as an indicator. The viral loads were reported as gene copies / mg of tissue, and presented as the mean viral loads plus standard deviation. Asterisks (**) indicate significant difference in viral loads (*p* < 0.01).

IBDV genome copy numbers in bursa of Fabricius samples of chickens from groups challenged with virulent IBDV, and genotype VII NDV genome copy numbers in the lung, trachea, spleen, duodenum, proventriculus, cecal tonsil, and bursa of Fabricius tissue samples of chickens from groups challenged with genotype VII NDV were determined by a SYBR Green I RT-qPCR. The detection limits of the RT-qPCR for IBDV and NDV were 1 × 10^4^ gene copies / mg and the results are presented in Figure 9A,B. Both IBDV and genotype VII NDV genome copies in the tissues of live rLaS-VIIF/HN-VP2 vaccinated chickens and the uninfected controls were significantly lower than those of the placebo controls. 

To examine whether vaccination with live rLaS-VIIF/HN-VP2 protected chickens from shedding challenge viruses, oropharyngeal and/or cloacal swabs sampled from each chicken on day one and day seven post challenge were examined for the shedding of genotype VII NDV and IBDV RNA by RT-PCR. Results in Table 1 showed that shedding of the challenged IBDV and genotype VII NDV ceased on day three post challenge in chickens vaccinated with live rLaS-VIIF/HN-VP2. However, placebo controls excreted IBDV and genotype VII NDV until death. 

## 4. Discussion

NDV and IBDV are two of the most important pathogens in poultry worldwide, causing tremendous economic losses to the world poultry industry. IBDV results in severe immunosuppression, which leads to vaccination failures and susceptibility to other infections and diseases [19]. Mixed infection of IBDV and NDV induces severe outcomes. The prevalence of novel IBDV variants and genotype VII NDV generates an urgent need of developing efficacious genetically matched vaccines. So far there is no commercially-available bivalent vaccine for controlling genotype VII NDV and novel IBDV variants. Recent studies indicated that live attenuated vaccines developed from a currently circulating genotype VII NDV using reverse genetics provided better protection than traditional vaccines. These genotype VII NDV-matched vaccine strains are identical to the circulating virulent NDV strains except for F protein cleavage site, which has been modified from a polybasic cleavage site to decrease virulence [11,12,13,14]. A variety of IBDV VP2-expressing viral vectors have been constructed and examined for the protection against IBDV infection [29,39]. These viral vectors are derived from live viral vaccine strains for other avian diseases. The resultant IBDV VP2-expressing viral vectors can be used as bivalent vaccines to protect chickens against two diseases. A couple of VP2-expressing viral vectors have been reported to confer protection in chickens against classical and vvIBDV successfully [2,39]. Currently some classical IBDV VP2-expressing herpesvirus of turkey vector is commercially available such as HVT-IBD and HVT-ND-IBD vaccine and used clinically to control Marek’s disease, ND and/or IBD. 

However, the evolution of the novel IBDV variants makes the previous IBDV vaccines no longer adequate. Therefore, in this study, a genotype VII NDV matched recombinant chimeric NDV LaSota vaccine strain expressing VP2 gene of a novel Chinese variant IBDV, rLaS-VIIF/HN-VP2, was constructed for the first time, in which both the F and HN genes of LaSota were replaced with those of the circulating and highly virulent Chinese genotype VII NDV strain FJSW. The cleavage site of the FJSW strain F protein in the rLaS-VIIF/HN-VP2 genome was mutated to the avirulent motif found in LaSota. Our results proved that one-dose inoculation with the rLaS-VIIF/HN-VP2 live vaccine generated remarkable antibody responses against virulent IBDV TL strain and genotype VII NDV. The HI titer against NDV (2^8^) and the ELISA titer against IBDV (>2000) reached the similar level as reported VP2-expressing NDV-vectored vaccines, respectively [27]. As the IBDV TL strain also has the typical amino acid residues of the variant IBDVs 249K and 286I, and can cause severe atrophy of the bursa of Fabricius and typical IBD, the rLaS-VIIF/HN-VP2 live vaccine can provide full protection against genotype VII NDV and variant IBDV. The efficacy of the rLaS-VIIF/HN-VP2 vaccine was demonstrated by blocking or reducing genotype VII NDV and variant IBDV shedding through oropharyngeal and/or cloacal routes (Table 1). Few or no gross and microscopic lesions in lung, trachea, spleen, duodenum, proventriculus, cecal tonsil and bursa of Fabricius tissues were observed in live rLaS-VIIF/HN-VP2 vaccinated chickens when compared to unvaccinated chickens. Correlation between NDV HI and IBDV ELISA antibody titers and live rLaS-VIIF/HN-VP2 vaccine efficacy was observed.

Novel variant IBDV has been identified as the etiological pathogen for the current epidemic of atypical IBD in China. The novel variant IBDV could severely damage the bursa of Fabricius of immunized chicken in the presence of antibodies induced by vvIBDV vaccines. There were obvious antigenic mismatches between novel variant IBDV and vvIBDV. There are typical amino acid residues in the hypervariable region (HVR) of VP2, and the key amino acid residues that might be involved in antigenicity and virulence differences of novel variant IBDV compared to vvIBDV were predicted [24]. Based on the fact that the VP2 protein contains major epitopes responsible for eliciting neutralizing antibodies [20], together with the results that one dose live rChLaSota-VP2 oculonasal vaccination induced full protection against IBDV lethal challenge and prevent the severe damages to bursa of Fabricius in present study, we proved that VP2 gene derived from the novel variant IBDV could be used as an immunogen for developing effective vaccine against IBD caused by IBDV variants and vvIBDV. The VP2 gene derived from the novel variant IBDV strain SHG19 has more than 96% sequence identity to the VP2 gene of other circulating novel variants reported in recent years, therefore, we believe that the constructed vaccine is able to provide protection against other IBDV novel variants as well. As the rLaS-VIIF/HN-VP2 vaccine provided full protection against genotype VII NDV, the chimeric NDV LaSota reverse genetics system could be used a platform for generating novel genetic matched bivalent vaccine rapidly for controlling mixed infections of different pathogens. 

Vaccination using live attenuated and inactivated vaccines is an effective way to prevent ND and IBD [39,40,41]. The limitation of inactivated vaccines is their inability to induce mucosal immunity. The protective efficiency of current live ND and IBD vaccines against genotype VII NDV and novel variant IBDV is not optimal due to the discrepancy of genotype of NDV and emergence of variant IBDV [42,43]. Live vaccines could be applied in chickens efficiently via the oculonasal route and induce both robust systemic antibodies, and mucosal and cell mediated immunity. This study indicated that recombinant genotype-matched live vaccine rLaS-VIIF/HN-VP2provided ideal protection against infection caused by genotype VII NDV and variant IBDV, and could reduce virus shedding and damage to bursa of Fabricius. The fact that immunized chickens showed reduced viral shedding in oropharyngeal and cloacal swabs after oculonasal challenge with genotype VII NDV and IBDV implied the live rLaS-VIIF/HN-VP2 vaccine induced local mucosal immunity. The reduction in shedding is also likely attributable to improved clearance and reduced peak viral load. Even though the viral determinants responsible for the virulence of NDV are not completely understood, the studies conducted to date suggest that the aa sequence at the F protein cleavage site determines whether an NDV strain is virulent (mesogenic and velogenic) or avirulent (lentogenic). But the degree of virulence of an NDV strain is determined by both envelope-associated proteins (M, F and HN) and internal proteins (N, P and L). Among all viral proteins, the F and L proteins play greater roles in determining the virulence of NDV [44,45,46]. As the LaSota-based genotype VII NDV matched vaccine in our system does not contain internal proteins (N, P and L) and envelope-associated M protein from genotype VII virulent NDV strain, the potential of reversion to genotype VII virulent NDV strain will be largely reduced as opposed to the genotype VII NDV virulent strain based genotype matched NDV vaccine by only modifying the sequence encoding the F protein cleavage site of virulent NDV from virulent polybasic (RRQKRF) to avirulent monobasic (GRQGRL).

## 5. Conclusions

In conclusion, chimeric LaSota with genotype VII NDV F and HN genes expressing novel variant IBDV VP2, rLaS-VIIF/HN-VP2 was constructed in this study. One dose of rLaS-VIIF/HN-VP2 live vaccine provided full protection against IBDV and virulent genotype VII NDV. These results indicate that rLaS-VIIF/HN-VP2 is an encouraging bivalent vaccine for controlling genotype VII NDV and novel variant IBDV. 

## Figures and Tables

**Table 1:** Shedding of challenged IBDV and genotype VII NDV in oropharyngeal and cloacal swabs.

Group	Challenge Virus	Survival Rate ^a^	Days Post Challenge ^b^													
			1		2		3		4		5		6		7	
			OP	Cloacal	OP	Cloacal	OP	Cloacal	OP	Cloacal	OP	Cloacal	OP	Cloacal	OP	Cloacal
rLaS-VIIF/HN-VP2	NDV	10/10	1/10	0/10	1/10	2/10	0/10	0/10	0/10	0/10	0/10	0/10	0/10	0/10	0/10	0/10
	IBDV	10/10		0/10		3/10		0/10		0/10		0/10		0/10		0/10
Unvaccinated	NDV	0/10	4/10	0/10	8/10	6/10	10/10	10/10	8/8	8/8	NS	NS	NS	NS	NS	NS
	IBDV	2/10		0/10		10/10	10/10	10/10		6/7		0/2		0/2		0/2

^a^ Number of survived chickens/total chickens; ^b^ Number of chickens with virus shedding/total chickens. OP, oropharyngeal; NS, no survivors.

## Data Availability

Data available on request from the authors.
